# Atomic Technologies and Nuclear Safety Practices in Spain During the 1960s

**DOI:** 10.1007/s00048-022-00335-w

**Published:** 2022-05-02

**Authors:** Ana Romero de Pablos

**Affiliations:** Instituto de Filosofía, Consejo Superior de Investigaciones Científicas, Madrid, Spain

**Keywords:** History of safety and radiation protection, Circulation of knowledge and atomic technologies, Zorita nuclear power plant, Franco regime, Spain, 1960s, Geschichte der Strahlensicherheit und des Strahlenschutzes, Zirkulation des Wissens und der Atomtechnologien, Kernkraftwerk Zorita, Franco Regime, Spanien, 1960er Jahre

## Abstract

The acquisition of a nuclear power reactor from the North American company Westinghouse in 1964 not only brought atomic practices and knowledge to Spain but also introduced new methods of industrial organization and management, as well as regulations created by organizations such as the US Atomic Energy Commission (US AEC) and the International Atomic Energy Agency (IAEA). This article analyzes the history of the knowledge, regulations and experimental practices relating to radiation safety and protection that traveled with this reactor to an industrial space: the Zorita nuclear power plant. Within this space, the appropriation, use, and coproduction of knowledge and practices were conditioned by political, economic, industrial and social factors, and by the engineers, researchers and other professionals who contributed expert knowledge. Material held in the Tecnatom Historical Archive—the engineering company that coordinated construction of the plant—is the main source for this work, which delves into the history of knowledge and atomic technologies and adds to the historiography of radiological protection in Spain.

## Abstract (de)

Mit dem Kauf des ersten Kernreaktors von der nordamerikanischen Firma Westinghouse im Jahre 1964 gelangten neue atomare Praktiken und Kenntnisse nach Spanien, ebenso wie neue Formen der Organisation und des Managements dieses Industriezweigs und bis dahin in Spanien unbekannte Regelungen, wie z. B. die der US Atomenergiekommission (US AEC) und der Internationalen Atomenergiebehörde (IAEA). Der vorliegende Artikel analysiert die Geschichte des – angeeigneten, genutzten und koproduzierten – Wissens, der Vorschriften und der experimentellen Praktiken hinsichtlich der Strahlensicherheit und des Strahlenschutzes, die mit der Inbetriebnahme des Reaktors im industriellen Komplex des Kernkraftwerks Zorita einhergingen. Dort, wo Wissen und Praktiken durch politische, wirtschaftliche, industrielle und soziale Faktoren bedingt waren, waren neben den Ingenieuren und Forschern auch andere Fachleute beteiligt, die gleichermaßen kompetentes Wissen beisteuerten. Das Material, das im Historischen Archiv von Tecnatom, dem Ingenieurbüro, das den Bau der Anlage koordinierte, aufbewahrt wird, ist die Hauptquelle dieser Arbeit, die versucht, die Geschichte des Atomwissens und der Atomtechnologien zu vertiefen und die Darstellungen zur Geschichte des Strahlenschutzes in Spanien zu erweitern.

Despite early evidence of the detrimental effects of ionizing radiation on health, the notion that radiation and its therapeutic use were harmless predominated in Western societies during the first half of the twentieth century (on the situation in Spain, see Herran 2008). When a private Spanish electricity company, Unión Eléctrica Madrileña (UEM), requested authorization from the Spanish Ministry of Industry to build a nuclear power plant in February 1962, the scientific, economic, and industrial authorities in Franco’s government were already keen to exploit the possibilities of atomic energy for civilian uses. A Spanish delegation that attended the First International Conference on the Peaceful Uses of Atomic Energy, held in Geneva in 1955, had been captivated by the new technology’s potential. The presence of Spanish politicians and industrialists at this international forum not only served to convince them that atomic energy was the way forward, but it also opened the door to Spain’s diplomatic—political and scientific—reintegration into the West, a situation that was utilized to construct domestic policies that reinforced the Francoist state (Romero de Pablos 2018a and 2019).

This fascination with the new technology in Spain left little space for consideration of the risks posed by ionizing radiation and the possible need for regulation (Menéndez-Navarro & Sánchez Vázquez 2013).

Before continuing, I would like to address an issue related to the constructed and non-neutral use of language. As those involved in producing historical scholarship are aware, conventions of naming and the meanings attributed to certain terms change depending on times and contexts. In the documentation explored for this article, North American directors, scientists and technicians have referred to the danger of exposure to ionizing radiation as a “hazard,” and not the “risk” that is much discussed in contemporary literature. The fact that these documents use the term “hazard” suggests changes that have to do with the technology itself and the risks revealed by its use, as well as the social perception that these same risks change over time: the first atomic accidents confirmed that such “hazards” could potentially cause harm to people and environment, and that indeed implied risks.

Use of the former term, with its closer connection to potential danger, and the shift to risk, that is, to the likelihood that it will cause harm, reveals the changes in social perceptions this technology underwent.

Conceptual debates on exposure to ionizing radiation—which at the time was nonexistent in Spain—took place in the United States between the mid-1940s and late-1970s. A significant shift in these conceptions happened at the start of the 1950s, when the term “tolerance dose,” used since the 1920s, was replaced by the concept of “maximum permissible dose.” This concept, which emerged from discussions at the Tripartite Conferences on radiation protection between scientists from the United States, Canada, and the United Kingdom in 1949–1953,^1^ referred to an amount that was not expected to cause appreciable harm to an individual over the course of their lifetime and demonstrated a degree of increased concern that swiftly moved beyond the space of scientists, physicians and x‑ray technicians (Mazuzan & Walker 1985: 38). This change reconfigured the concept and scope of radiation protection: it shifted from a medical and industrial problem of limited proportions to a major public health issue. New units of measurement that reflected these concerns were introduced: “rad” indicated the amount of radiation absorbed by a person (that is, the amount of energy deposited in human tissue by radiation); “rem” denoted the biological risk of exposure to radiation. The National Committee on Radiation Protection (NCRP) in the US published *Maximum Permissible Amounts of Radioisotopes in the Human Body and Maximum Permissible Concentrations in Air and Water* in 1953. This guide not only echoed the work of geneticists such as Hermann J. Muller, the 1946 Nobel Prize winner in Physiology or Medicine who had reported the vulnerability of cells to even low levels of radiation, but also foreshadowed the main focus of concern during the mid-1960s: these centered on the hazards of radioactive effluents released from nuclear power plants (Walker 2000). *Physics Today,* published by the American Institute of Physics, declared the NCRP’s guide essential for three reasons: 1) it included details of methods for estimating maximum permissible concentrations; 2) the factors determining radioisotope hazards were made visible; and 3) recommendations were made on how to avoid or at least minimize these hazards (Permissible Radioisotope Concentrates 1953: 14).

It is likely that this guide was included in the library on atomic issues that US Ambassador John O. Lodge presented to the president of the Spanish Nuclear Energy Board (JEN)—an agency used to secure state control over all processes relating to atomic energy (Romero de Pablos & Sánchez Ron 2001)—in November 1955, in the hope that future knowledge and research on nuclear development between the two countries would be exchanged (Romero de Pablos 2018b: 63–84). Similar libraries had been presented as gifts to all countries that signed partnership agreements with the United States through the Atoms for Peace campaign, as Spain had done in July 1955. Brought to fruition by Admiral Lewis Strauss, president of the US AEC, and the Spanish Ambassador to Washington, José María de Areilza, this agreement established the framework for interaction with the United States in all matters relating to nuclear research.^2^

While this agreement provided a legal basis for Franco’s regime to demonstrate that they shared the promises and opportunities of civilian atomic energy use with the United States and the West in general, it also presented new possibilities for the US to sell and test materials and technology (Creager 2002 and 2013; Krige 2006 and 2010; Oreskes & Krige 2014; Drogan 2016).

It is in this context that I analyze the history of knowledge, regulations, and experimental practices for radiation protection and safety that were developed, appropriated, used, and coproduced by Spanish engineers and technicians. The acquisition of a research reactor from the General Electric Company in 1958 (Romero de Pablos & Sánchez Ron 2001: Chapter 2) and other power reactors as of 1962 (Rubio-Varas & De la Torre 2017: 250–254; Romero de Pablos 2019) required the JEN and the electric companies to train researchers with new technologies. Within the framework of the cooperation agreement that accompanied the sale, these employees were able to travel to the United States for laboratory training (Barca Salom 2010; Soler Ferrán 2015; Rubio-Varas & De la Torre 2018: 85–110).

One field of research prompted by the arrival of atomic technology in Spain was nuclear safety, which began to take shape in the late-1950s. The JEN chemist Agustín Alonso was one of the first Spanish researchers to enter this new disciplinary space by attending the initial course on Nuclear Reactor Hazards Evaluation held at the Oak Ridge School of Reactor Technology (ORSORT) in 1959. Alongside students from Belgium, Brazil, China, Denmark, Germany, Great Britain, Israel, Italy, Japan, Pakistan, the Philippines, Switzerland, Vietnam and the United States, he attended regular lectures on various subjects related to reactor analysis, such as heat transfer, shielding, health physics, instrumentation and control, experimental physics, materials, engineering, and stress analysis. Agustín Alonso’s year of training also included study of meteorological and geological considerations in reactor location, plus waste disposal and the accidental release of fission products. The course emphasized the heat transfer problems of fuel elements and coolants, reactor kinetics and containment, and the probabilities of incidents and risks involving both equipment and public safety. Supplementary lectures were provided by various guest lecturers. After nine months of classroom lectures, the students gained practical experience and knowledge through a three-month study project, designing and completing a hazard report (Oak Ridge School of Reactor Technology 1959: 32–34).

On his return to Spain, Agustín Alonso brought the knowledge he had acquired at Oak Ridge to the embryonic JEN safety group. As JEN Director of Nuclear Safety until 1980, he was responsible for creating and developing the Spanish Regulatory System for nuclear power plants. Some knowledge and work practices were initially tested on the research reactor, then developed and expanded throughout the various nuclear plants across Spain. Although dependent on the particularities imposed by the distinct technology arriving in Spain, these abilities and skills created habits and standards that define the history of radiation in Spain. The accumulation of this theoretical and practical knowledge also permeated academic research and teaching: Agustín Alonso was a Professor at the Barcelona (1976–1981) and Madrid (1981–2003) Polytechnic universities.

As the construction of nuclear facilities expanded across Spain, the JEN safety team also expanded exponentially.^3^ Charged with preparing reports for the Ministry of Industry on requests to build radioactive facilities and the transportation of domestic radioactive devices and substances, the group was organized into four departments: evaluation, inspection, regulations, and sites. This suggests that the arrival of technology from the US, and subsequently from France and Germany, prompted not only a division of labor for JEN researchers and technicians, but obligatory specialization. The PWRs (Pressurized Water Reactor) and BWRs (Boiling Water Reactor) manufactured by, respectively, US companies Westinghouse and General Electric, were the dominant reactor types in Spain, with six PWRs and two BWRs. One UNGG type reactor (Natural Uranium Graphite Gas) with French technology and a PWR manufactured by the German KWU (Kraftwerk Union) were also installed. Political and economic considerations, rather than technological ones, prompted the introduction of these European technologies into a country that would become one of the biggest clients for the North American atomic industry.

The French graphite moderated, natural uranium gas cooled reactor was significantly more expensive than the US products, and this selection for the Catalan power plant Vandellòs (1972) highlights the dictatorship’s political eagerness to forge nuclear industrial links with the neighboring country. The agreement to exchange energy and nuclear cooperation that came about through a consortium of Catalonian electrical utilities and Electricité de France enabled the French state to export a technology they already deemed obsolete and the Spanish government to appease Francoist families who, rooted in autarkic policies, advocated for the use of domestic natural uranium (Sánchez-Sánchez 2017).^4^ Likewise, UEM’s purchase of a KWU type reactor for the Trillo power plant cannot be understood without taking into account the industrial, diplomatic and personal relationships developed between Spain and Germany as of the end of the 1940s (Presas 2000 and 2005). This, along with the creation of a complete fuel cycle nuclear industry in West Germany, endorsed the KWU technology (Sanz Lafuente 2017). Although it goes beyond the limits of this work to delve deeper into the security practices that French and German technology introduced in Spain, it is logical to assume that, just as with North American technology, Spanish technicians had to educate themselves, confront new experiences, and also learn other skills that were required by the new technologies.^5^ The documents preserved suggest that the ongoing contact maintained by the JEN’s Nuclear Safety Department with the analogous French and German departments allowed Spanish technicians to assimilate other knowledge and to standardize practices. These contacts should also be understood as a result of the diplomatic and political movements made by the Spanish state to present itself as modern, connected to multinational organizations and regulatory institutions from other countries.

The 1964 Law on Nuclear Energy also came into being as a result of transnational pressures and influences.^6^ Establishing a legal framework for the development and implementation of civilian applications of nuclear energy in Spain, the law included the path already traveled—with the exception of the 1959 order on ionizing radiation,^7^ virtually free from regulation—since creation of the JEN in 1951. The Organisation for European Economic Co-operation (OEEC) had expressed the wish that all member countries should adopt the necessary measures to guarantee adequate protection against the hazards of ionizing radiations for both for workers and the general population; the OEEC thus called upon the member countries to ensure that appropriate measures were in place in the event of emergencies or accidents involving ionizing radiation.^8^

The 1964 law provided basic regulatory principles and declared all nuclear activities and use of radiation sources to be risky and therefore subject to regulation, with the Ministry of Industry and Energy as regulatory authority and the JEN as the technical body responsible for evaluating the safety of any application and inspecting all nuclear installations and activities utilizing radiation. The law also established the principles to be followed to secure radiological protection, along with third party liability and the corresponding compensation. Responsibility for safety was assigned to the licensee and provided the basis for sanctioning any misconduct. So, on the one hand, this law addressed obligations arising from international agreements that required regulation of civil liability in the event of nuclear accident and coverage of risks associated with this industrial sector, and, on the other, the responsibilities imposed by the technology itself.

Along with new atomic practices and knowledge on safety and radiation protection, Spain was now subject to novel regulations and industrial organization. I argue that all of this *knowledge in transit*—following James Secord’s conceptualization (Secord 2004)—participated in the construction of a new national and international political narrative: from the outset of the 1960s, the Francoist State was an internationally connected nation capable of incorporating the latest atomic technologies.

This new discourse enabled the Franco government to rewrite existing geopolitical and diplomatic histories (Roqué & Herrán 2013) and participated in construction of North American technological hegemony (Krige 2006). The reactor was viewed by Spanish engineers and the electricity industry as a hybrid of technology and politics (Hecht 2009), a tool for facilitating political negotiation and shaping the sociopolitical order of the Spanish nation.

For this reason, I propose analyzing and exploring atomic practices in Spain, and specifically radiological protection, as a highly political, ideological, and diplomatic endeavor that needs a transnational and interdisciplinary approach.

## Zorita Power Plant

In February 1962, UEM agreed to request authorization from the Ministry of Industry to build a nuclear power plant. Two circumstances led to this decision: the capacity of the region’s rivers to generate hydroelectric power had been exhausted, and studies by the company itself and the Ministry of Industry predicted an increase in demand for energy during the 1960s and 1970s. At the beginning of the 1960s, all of UEM’s electricity production facilities used water, thus it was clear they needed to invest in alternative sources; the question was whether to opt for a classic thermal power plant using oil or coal, or a nuclear power plant.

The Spanish electricity company had noted the 1958 decision by the North American company Pacific Gas & Electric to expand its thermal power plant in Humboldt Bay (San Francisco) with a third 60 MW unit powered by a boiling water nuclear reactor (BWR). According to the US Company, it was cheaper to build a nuclear facility than one that used oil as fuel.

It was then that UEM asked Tecnatom (Técnicas Atómicas, S.A.), an engineering research company created in 1957 by the main Spanish industrial bank, Banco Urquijo, to determine which of the two options, conventional or nuclear, was more suited to its interests. The head of the company that would soon become one of the central players in Spanish nuclear industrial development was Jaime Mac-Veigh Alfós. The Irish-born industrial engineer, who had travelled to the United States between 1948 and 1950 to study the construction and startup for the TALGO light rail train (Tren Articulado Ligero Goicoechea Oriol) in Spain,^9^ would become a major actor in the Spanish nuclear program: in addition to being one of the leading figures in the Zorita Project—he negotiated import licenses, dealt with the logistics to transfer the reactor, negotiated loans with public and private North American banks—and the person behind the Spanish nuclear lobby that promoted the creation of the Spanish Atomic Forum (1961), he also indirectly contributed to a turn in industrial policies that left the business of nuclear power plants in Spain in the hands of private companies (De la Torre & Rubio-Varas 2018: 113 y 123).

Mac-Veigh returned from the US convinced of the possibilities offered by energy generated from the fission of uranium (De la Torre & Rubio-Varas 2016: 4–5; De la Torre 2017: 41–42). In a study published in 1957, he insisted that Spain would face a significant deficit in conventional energy by 1970 and estimated the nuclear power that Spain required to counter this at 350 MW (Mac-Veigh 1957: 93). Mac-Veigh’s predictions were borne out in 1971 with the startup of Santa María de Garoña (Burgos), the second nuclear power plant connected to the Spanish electricity network.

The UEM request prompted Tecnatom’s engineers to make several trips to San Francisco to study the technical and industrial features at Humboldt Bay.^10^ They met with managers of Pacific Gas & Electric, owner of the power plant; with the main contractor, Bechtel Co.; with General Electric, supplier of the nuclear equipment; and with other American firms capable of exporting techniques and supplying equipment for nuclear power plants. The engineers realized that Humboldt Bay was not an isolated case and that many other companies were in a position to make competitive bids. All this convinced UEM of the possibilities of nuclear power and the benefits of constructing a plant in Spain.

In 1962, when Tecnatom technicians were conducting a preliminary report on the type of nuclear plant to build, 22 power reactors were operating across the world, only four of which were cooled by water.^11^ One of the first decisions UEM made was to select the fuel type. The choice between natural or enriched uranium in Spain went beyond scientific and technical discussions. It also represented a major political issue that had long concerned and divided those responsible for Spanish nuclear policy: on one side were the most conservative people who, in accordance with autarkic policies, championed the use of a reactor fed with natural uranium from Spanish mines; on the other were the technocrats, convinced that Spanish nuclear development was not possible without international support and foreign technology.^12^ Finally, following numerous reports by Tecnatom engineers, the UEM decided on a water cooled reactor fed by slightly enriched uranium. This decision not only imposed dependence until 1969 on the US AEC, the only organization authorized to enrich natural uranium, but also limited the choice of technology to two options: PWR or BWR (Walker 1992).

In February 1964, following a long tender process, a group of expert technicians from UEM, Tecnatom, the JEN, and a North American engineering consultant, the Bechtel Corporation, led by Manson Benedict, Professor of Nuclear Engineering at the Massachusetts Institute of Technology and chair of the US AEC advisory committee, decided the tender best suited to UEM’s interests was the one submitted by Westinghouse. This tender process was more than a tool to bid for the nuclear power plant: it enabled UEM to convince the dictatorship of the viability of its project, and to present itself before the leading North American nuclear reactor manufacturers as a modern company that acted in accordance with international competitive practices. The decision to put the tender under the direction of Manson Benedict not only demonstrated the Spanish electricity company’s international networks and connections with high level nuclear utility company consultants, it also emphasized UEM’s willingness to accept standards or regulations originating from the US AEC.

Unlike the three other tenders, from Allis-Chalmers Manufacturing Company, Combustion Engineering, and General Electric, Westinghouse proposed that UEM opt for a closed cycle PWR reactor with a net power of 153.2 MWe, similar to the Yankee Rowe power plant put into operation in 1960. The engineered design established a complete separation between the nuclear reactor and turbine plant, a common feature of closed cycle water reactors, which not only provided greater safety but, as the secondary system could be operated independently, allowed for later expansion. Another influential factor was that these reactors could detect the existence of radioactive fission isotopes, mainly radioactive isotopes of iodine, in the water of the primary cooling system, thereby warning of any rupture in the fuel cladding. This directly affected the safety of the facility and, as we will see with the installation of the reactor in Zorita, led Spanish technicians to introduce this condition into their protocols along with other practices intended to control the release of radioactive substances.

The technical and safety features offered by the Westinghouse reactor were important in its selection. But they were not the only reasons. Selection of the Westinghouse proposal was not only technical, but also political. The first Spanish nuclear law (1964, see annotation 4) stated that at least 40% of nuclear plants had to be built by local companies,^13^ and the Westinghouse project allowed a higher degree of participation by local technicians than the other proposals.^14^ However, it is important to note that Spain produced uranium, and the agreement was linked to negotiations with the US AEC to enrich Spanish produced uranium in the United States.

UEM and Westinghouse signed the contract in December 1964. By then the North American manufacturer had supplied its technology to three power plants already in operation: Shippingport and Yankee Rowe in the United States and Selni in Italy. It was also the supplier for three plants under construction: the Sena plant—a joint Franco-Belgian project—in France, and the San Onofre and Connecticut plants, both in the United States.

On July 5, 1965, civil engineering began on the Zorita nuclear power plant. Still, knowledge other than atomic physics and chemistry had come into play in order to reach this moment. I argue that the detailed hydrological, geological and meteorological studies undertaken to determine the location of this plant created patterns and working practices that also participated in the standardization of radiation safety in Spain.

## Safety and the Placement of the Zorita Power Plant

As of the end of the 1950s, the close connection established between the location of nuclear reactors and assessments of their safety led to increased attention to site exploration as a subject of study.

In the early years of commercial atomic power reactor development, the US AEC was faced with the task of determining technology-based standards for regulating private industry that met its twin policy objectives: promoting the industry and protecting the public. Although setting parameters to guide the industry in the location of nuclear reactors had been a priority for the US AEC since the early 1950s, agreements were often lengthy and contentious procedures. Following protracted technical negotiations between US AEC regulatory staff, the Reactor Safeguards Committee and, of course, the industrial community, the Commission approved site criteria in 1962 (Mazuzan & Walker 1985: 214–246).

Although isolation had initially been one of the most crucial criteria in regulating the placement of nuclear facilities, the power of the electrical industry, which wanted to install plants close to potential customers, led to a reevaluation of the very concept of isolation. In this sense “the AEC assumed that their criteria incorporated an ample margin of safety while at the same time allowed for the industry view that commercial power reactors should be located close to population centers where the greatest demand for electricity existed” (Mazuzan & Walker 1985: 243–244). As we will see in the next section, this required regulators to place greater emphasis on engineered safety features and to continue making site evaluations on a case-by-case basis.

Research on the location of nuclear research centers and power reactors was increasing. Aware that this was a controversial issue, the IAEA convened the first symposium on the subject in Bombay (now Mumbai) in 1963, with the aim of reaching a consensus on guidelines for site selection and conveying to the public that sites were only chosen after exhaustive scientific assessment. Discussions at this meeting principally covered four major areas: 1) environmental considerations with particular reference to atmosphere and the ground; 2) containment as it affected site selection; 3) criteria for site selection; and 4) experiences relating to site selection for nuclear research centers and power reactors. Proceedings from this symposium, gathered under the title *Siting of Reactors and Nuclear Research Centres*, show that, along with geological conditions and engineering, other requirements that made site selection a complex and delicate issue were also discussed: population, consumption centers, access, climate, and the presence of water for cooling. This complexity increased when social problems began to arise. Although the social discontent that nuclear power plant facilities were starting to produce was briefly discussed at the Bombay meeting, remarks were often cautious and the issue went largely unaddressed. To combat this, signatories to the symposium proceedings, who were attached to the Health and Safety Branch of the United Kingdom Atomic Energy Authority and to the Electricity Division of the UK Ministry of Power, recommended educating the public to be “capable of assessing nuclear hazards realistically and objectively” and to avoid extremes, whether excessive anxiety or extreme complacency (IAEA 1963: 343–354).

This symposium was one of the first forums where researchers and technicians began to problematize the location of nuclear reactors. The conclusion reached echoed that expressed by US AEC regulatory staff: establishing clear criteria to determine the suitability of sites would be extremely difficult. The complexity of the problem, coupled with industrial interests, prevented the analysis process from being standardized. For this reason, each nuclear facility had to be subject to specific assessment.

Although there was no Spanish representation at the Bombay meeting, the same concerns appeared in the first location reports commissioned by UEM. The experience and knowledge acquired by Tecnatom’s technicians during their trips to the United States, and the practices the JEN safety group had adopted as their own, led to observance of US AEC regulations as well as attention to issues and concerns regarding research on nuclear reactor sites. Spanish researchers and technicians were familiar with the literature showing geology, topography, meteorology and hydrology as important elements in evaluating and selecting a reactor site (DiNunno et al. 1962). And the program, which at the request of the US AEC had been designed by the Weather Bureau Office at Oak Ridge between 1948 and 1952, was an important reference (Holland 1953). Focusing on knowledge related to observation techniques, microclimatology, in this case of the southern Appalachian Valley, and the relationship between stability, wind flow, turbulence and diffusion in hilly terrain, this study was taken into account by the Spanish technicians. Therefore, one of the first actions UEM took after deciding to build a nuclear power plant was to request that the Geological and Mining Institute of Spain conduct a hydrogeological study of an area in the central province of Guadalajara. With this study, UEM sought to learn about the depth and trajectory of radioactive water, which could infiltrate into the ground in the event of an accident and if the power plant’s deposits were ruptured.^15^

The topography of the area was defined by a plateau and the Altamira mountain range, with clear geological variations. The plateau consisted mainly of tertiary soil, gypsum, sandstone and limestone. The hard cretaceous limestone of the mountain range had resisted the erosion of a hard and dry climate with extreme seasonal temperature fluctuations. The area as a whole had little vegetation and agriculture was scarce. According to the study, only reservoirs built on the Tagus River during the 1950s provided the area with any industrial potential.

After probing to depths of up to 60 meters, measuring the ionic change capacity of the most typical terrain in the area and testing permeability, engineers from the Geological and Mining Institute told UEM the land was geologically and hydrologically suitable, with one condition: the reactor vessel and radioactive wastewater deposits had to be installed below the lowest level of the riverbed. This, they concluded, would prevent radioactive waters from the plant entering the Tagus River in the event of an accident.

Along with the hydrogeological study, UEM also took records and measurements of air temperature, wind speed, and wind direction into consideration. To this end they installed a 75m high meteorological tower, equipped with sensors at 2.35 and 75m. This report, commissioned from EPTISA (Industrial Technical Studies and Projects S.A.), also incorporated data from the Barajas observatories between 1950 and 1954, Alcalá de Henares from 1958 to 1960, the Guadalajara Meteorological Institute from 1951 to 1960, and the Getafe Air Base between 1958 and 1962. This data was useful for predicting the behavior of radioactive gases released during both normal operation of the type of power plant they proposed and in the event of an accident. The report concluded that, from a meteorological perspective, the site did not present problems for a reactor with the projected specifications.^16^

The two reports convinced UEM that Almonacid de Zorita (Guadalajara), on the left bank of the Tagus River and on the edge of the Zorita reservoir, was the best place to install the first nuclear power plant in Spain. Additional features included the availability of sufficient water to cool the plant and its accessibility by road and railway. The site was also only 65 km from Madrid, a major consumption center due to population and industry, 40 km from Guadalajara and 70 km from Cuenca. The fact that there was no densely populated hub within 30 km—nearby residents were concentrated in small towns—was also a determining factor.^17^ Undoubtedly the most important factor was that central Spain was UEM’s most significant market. The area had a high growth rate and, as mentioned, all data indicated that energy demands would be extremely difficult to meet due to a lack of fossil fuels and exhausted water resources.

The debate over siting criteria occupied a space where, on the one hand, Spanish technicians and researchers as well as Spanish politicians sought to protect public health and safety while, at the same time, the sought to actively develop this new industry. The US AEC regulators, and with them other countries like Spain that purchased North American technology, thus assumed that the criteria incorporated a wide margin of safety while also taking into account the industry’s view that commercial power reactors should be located close to population centers where there was the greatest demand for electricity.

In 1967, four years after the meeting in Bombay, the IAEA organized a second symposium in Vienna. By this time nuclear power plants had become newsworthy and their complex construction processes took centerstage. In the IAEA’s view, this development required new discussion forums to reach a consensus on working practices and criteria.

During the meeting, IAEA officials proposed that, in order to avoid the possible consequences that leakage of radioactive products could have on public health and safety, site selection, the reactor project and its inherent system safety, and engineered safeguards needed to be developed simultaneously (IAEA 1967). Thus, the suitability of a site required justification through a critical study of possible accidents and analysis of the containment capacity provided within the nuclear power plant design. While this procedure, which connected the reliability of sites with the reliability of technologies, had been under testing, it had taken some time to incorporate technical accounts that connected types of accidents with their radiological effects and subsequent performance protocols.

The relationship between remote siting, engineered safeguards and protection of the public had long concerned the US AEC Advisory Committee on Reactor Safeguards (ACRS). In 1964, Herbert Kouts, chairman of the ACRS, told Glenn Seaborg: “The protection of the public ultimately depends on a combination of engineered safeguards and adequate distances. Engineered safeguards which can justify decrease of the distances must be extraordinarily reliable and consistent with the best engineering practices as used for applications where failures can be catastrophic” (Walker 1992: 141).

In the case of Zorita, two such accounts were included in the preliminary safety studies commissioned by UEM in 1963 and written by Westinghouse, the technology supplier, and the Tecnatom technicians (Westinghouse 1964). Both reports suggested that it was the technology and its implementation that initiated safety practices, established parameters that needed to be respected, and dictated a course of action in the event of an accident.

It is not surprising, therefore, that in the work presented by Agustín Alonso and other members of the JEN safety group at the meeting in Vienna, they first used the concept of “reference plant” (Pascual et al. 1967). This term refers to a nuclear power plant that is already in operation and whose design serves as a guide to draw up plans and construct another. All the information and documentation that is useful to justify and approve the reference plant is often later used by decision-makers to concentrate on examining possible differences and thus facilitate the selection process of the technology.

When Zorita construction began in 1965, there were three power plants in operation with Westinghouse technology: Shippingport and Yankee Rowe in the United States and Selni in Italy. Although Zorita did not have a reference plant as we understand it today, its construction had a precedent in the Yankee Rowe plant set up by Yankee Atomic Electric Company in 1960 that used Westinghouse as a reference to convince the technicians from UEM, Tecnatom, the North American consultant Bechtel Corporation and the JEN that their technology was the best option.

Curiously, Zorita would become a reference for the power plants that were later built in Spain. Although with different technology, the know-how and practices that the Zorita technicians had internalized about radiological protection turned them into the main agents for much of the knowledge that made what came later possible.

The “reference plant” concept perfectly evokes the situation of Spanish nuclear facilities built on “turnkey” contracts: the reactor manufacturer provided the majority of the engineering. One of the general principles in nuclear safety is that of proven engineering: the design, construction and operation of a nuclear power plant must be based on established and tested engineering practices. This principle was particularly significant for importing countries such as Spain. With technology came the codes and standards used in engineering design and, of course, safety and radiation protection regulations. It is within this context that the concept of “reference plant” must be understood. Although the inherent system safety practices had been designed and tested for each technology in their countries of origin, upon arriving in Spain and being reproduced and used as their own, the heterogeneous Spanish nuclear facilities were normalized and standardized according to the technologies and reference plants (for a complete list of planned nuclear power plants in Spain, see Rubio-Varas & de la Torre 2017: 249–254). As stated, economic, industrial and, above all, political reasons conditioned the choice of reactors in each power plant and, consequently, safety practices and the history of radiological protection in Spain (Romero de Pablos 2019).

## Safety and Engineering at the Zorita Power Plant

A 1967 report by UEM for the Ministry of Industry expressly stated that the safety guidelines followed at Zorita were in response to “the safety characteristics intrinsic to the type of pressurized water reactor” acquired from Westinghouse (Unión Eléctrica Madrileña 1967). For both UEM and the Ministry, incorporating safety practices that had been tested and proven in other facilities with the same type of reactor—Shippingport and Yankee Rowe in the United States and Selni in Italy—was a guarantee that any potential risks were minimized. The principal danger in nuclear power production was a malfunction in coolant circulation, leading to the reactor overheating and releasing radioactive material or fission products into the environment. By the mid-1960s, this was the main issue of concern for safety experts.

A complete separation of the nuclear reactor and turbine plant, characteristic of closed cycle water reactors such as the 153.2 MWe PWR installed at Zorita, increased safety. In addition, as stated, these reactors could detect the existence of radioactive fission isotopes, mainly radioactive isotopes of iodine, in the water of the primary coolant system, indicating a rupture in the fuel cladding. As I have already described, these two safety conditions provided by the reactor’s engineering helped convince UEM decisionmakers that this was the most suitable technology. Other safety conditions also traveled with the power plant design. These were subjected to critical studies to evaluate the radiological effects that various accidents could produce and thus to assess the system’s containment capacity.

Zorita had four containment barriers. The first was provided by the intrinsic retention properties for fission products of the uranium dioxide itself. The others were the fuel cladding, the reactor’s coolant system, and the containment enclosure. The latter was a semi-spherical steel dome, 31 m in diameter, resting on a cylindrical wall of reinforced concrete 34 m high and almost 1m thick, which in turn was supported by a deep foundation of concrete. This space, built to house the reactor vessel, the boiler or steam generator, the main pump, and the pool to store spent fuel, structured the architectural assembly of the plant. These four barriers formed the basis on which the power plant’s safety principles were established.

Studies on the possible failure of these barriers, as detailed in safety reports, considered an uncontrolled release of radioactivity to be a real possibility. To attempt to ensure that any barrier failure could be borne without serious safety consequences, UEM and Westinghouse established specific protocols of action for various hypothetical accidents.

But before continuing with the hypothetical accidents tested at Zorita, and with the aim of situating the long and complex debates that were held on nuclear safety at the end of the 1950s and beginning of the 1960s, it is of interest to pause for a moment on the concept of “maximum credible accident.” Proposed by Clifford Beck, Deputy Director of Regulation for the US AEC, this represented the intermediary solution that brought two distinct and distant ways of understanding the problem closer together: if only the worst imaginable accidents were considered, then only sites hundreds of kilometers away from populated areas could be considered suitable. On the other hand, if sufficient safety features were included in the reactor design to protect it from the worst imaginable accidents, any site could be acceptable. Both positions seemed unrealistic, so the regulators found a middle ground: the maximum credible accident. But the debates did not end here. Only taking into account the maximum credible accident could lead to neglecting less serious accidents. This is why the US AEC proposed requesting, from those responsible for the technology and from plant owners, identification of the various types of credible accidents for the specific type of reactor they proposed to set up (Mazuzan & Walker 1985: 229). And this is how they proceeded at Zorita.

Situations that could lead to reactivity accidents included issues when starting up the reactor, the accidental removal or expulsion of control rods with the reactor in operation, and problems with the boron concentration used as a moderator.^20^ Studies were also conducted on mechanical accidents such as loss of coolant, incidents with fuel handling, steam pipe breakage, a loss of feed water flow, the rupture of steam generator pipes, and accidents caused by load loss.^21^ These reports also identified potential problems resulting from a loss of electrical power and from fire.

In order to estimate the possible extent of radiological effects, the safety studies used as a hypothetical reference a total breakdown of the primary cooling circuit with a simultaneous failure of the two independent safety circuits. To establish hypotheses and proceed with analysis, the researchers used criteria published by the US AEC in the Technical Information Document (TID) entitled “Calculation of Distance Factors for Power and Test Reactor Sites,” known as TID-14844. This report outlined a methodology for calculating radiological risks and included guidance on assumed fractional release to containment, atmospheric transport and dispersion behavior, and the calculation of offsite consequences (US AEC 1962). Following this methodology, Spanish technicians estimated emission speed values for the first 24 hours and up to a period of 30 days in unfavorable weather conditions. The results of this hypothetical accident study concluded the nuclear power plant could operate without undue risk (Pascual et al. 1967: 97).

However, as I will show, all the situations recorded in the reports were based on assumptions and theoretical values that were adjusted and, in some cases, corrected by the initial startup of the reactor. And although this was normal in engineering, the different tests that were done to put Zorita into operation showed the Spanish technicians the need to revise calculations and values that had been previously tested and taken as valid, while also showing the North Americans the difficulty of standardizing certain safety criteria that required specific and continuous updating.

The first experimental values from December 1967 were obtained with zero power testing at the plant.^22^ These tests, in addition to educating and training plant technicians, detected deviations between the theoretical and experimental parameters. For example, measuring the temperature coefficient of the moderator with different boron concentrations and various rod configurations was one of the safety techniques introduced by the reactor technology. Zorita technicians learned to vary boron concentrations by compensating for changes in reactivity by moving the fuel rods. As the experimental values were slightly lower than those drawn up by Westinghouse, the technicians introduced their own corrections.

The first core loading took place in March 1968. Plant engineers followed guidelines in *The Nuclear Design of the Zorita Reactor*, in which Westinghouse established the configuration of the reactor core. An account of this process by plant engineers indicates some of the parameters did not match those specified by Westinghouse. This was the case, for example, with the boron concentration. The presence of a higher or lower concentration of boron is important because, by absorbing neutrons, this chemical element acts as a moderator and can be used to control and, if necessary, stop the nuclear fission reaction.

Another experience Zorita technicians underwent during that initial core loading was closely related to one of the risk situations contained in the Westinghouse safety reports. Although personnel had been trained in the use of tools to handle fuel, one of the elements was irreparably damaged when it became detached from the handling machine and plunged into the irradiated fuel pool. The documentation does not clarify whether this was human or mechanical error—stating only that during the transfer process from the tool hanger, the fuel element provided with the control rod disengaged and fell—but was probably a combination of the two. There was no release of radioactivity, as the damage did not affect the cladding; however, as the two fuel elements in reserve were highly enriched, not lowly enriched like the damaged one, the core configuration required adjustment. Following accident analysis, UEM and Westinghouse engineers physically modified the fuel handling tools, introduced loading methods that were different from those recommended by Westinghouse, and trained staff in this new practice.^23^

The Franco dictatorship’s determination to use uranium extracted from Spanish mines for the first reactor core at Zorita had led them to negotiate with the US AEC for its isotopic enrichment and with two North American companies: Allied Chemical for the conversion of Spanish uranium into natural uranium hexafluoride, and Westinghouse for the manufacture of fuel elements. Thus, in 1966, 137 tons of uranium ore was transported from the Port of Cadiz to the Port of New Orleans. From there, the uranium ore traveled to the US Department of Energy sampling station at Grand Junction in Colorado for the uranium concentration to be checked and calibrated. Allied Chemical processed the Spanish uranium to obtain uranium hexafluoride, a necessary prior step to its isotopic enrichment on US AEC premises. Following manufacture of the fuel elements at the Westinghouse factory in Cheswick, Pennsylvania, the ready-to-use enriched uranium returned to Spain.

When an accident with the fuel element happened, Zorita technicians had to modify fuel distribution in the reactor core with what was available: the 71 fuel elements—69 for the initial core load and two spares—that had arrived at the Port of Bilbao between November and December 1967 following the journey through the United States.

The nuclear tests intended to bring the reactor to critical state in a safe and controlled manner made Zorita a space for the creation of knowledge that, in addition to being used to construct and operate other nuclear facilities, also opened up spaces in academic and industrial teaching and research. During its 38 years of activity, Zorita was a laboratory in which materials, practices and knowledge about radiological safety and protection were tested. Much of this knowledge produced unprecedented disciplinary spaces in Spanish universities. Among many others, the professional and academic career path of Agustín Alonso is one example. Zorita was also a training ground and learning space for engineers and technicians for future nuclear power plants.

Appropriation by Zorita technicians of the various practices imposed by the Westinghouse reactor, and the new knowledge they developed based on these practices, led to UEM, Westinghouse and the JEN launching a joint research project in 1969 on the behavior of fuel rods in the presence of very high radiation (Zorita Research and Development Program 1969). This project required the JEN to build the “Hot Cell of a Thousand Curies,” named for the level of interior radiation it could attain. In this facility, the agency created by the State to control atomic research in Spain prepared samples to send to Westinghouse laboratories. This project enabled a Spanish industrial power plant—Zorita—and a Spanish research center—the JEN—to work together to understand the behavior of fuel elements, introduce improvements, study waste management, and consider the possibility of reprocessing for the first time. None of this would have been possible without the reactor’s arrival.

On June 30, 1968, the Zorita reactor achieved its first criticality. On July 17, three years after the start of construction, synchronization with the Spanish electricity grid took place with the Minister of Industry and the media in attendance. But the official inauguration did not take place until December 12 of that year, when Francisco Franco himself toured the facilities, accompanied by the Vice President, the Minister of Industry, and the president of UEM’s Board of Directors (ABC 1968; La Vanguardia 1968). Among the wide media coverage, the *ABC* newspaper hailed the new “atomic light” while the Official Newsreel *No-Do* focused on the Spanish origin of the uranium, alluding to the autarkic policies that many still supported.^26^

On July 17, 1968, the plant became operational. Of the “turnkey” facilities exported by the United States, Zorita was the first to be connected to a national network.^27^

## Conclusions

When Franco’s government authorized a Spanish electrical company to build the first nuclear power plant in the country, a significant change was ushered into the history of Spanish radiation protection. Until that time, atomic research had only been conducted by a state organization—the JEN—under strict state control. Although this agency had established a number of pilot facilities, the political decision to produce nuclear based electrical energy in Spain opened up a new space for radiation safety and protection.

The history of radiation protection in Spain, as in other countries, goes beyond national borders. With the arrival of atomic technology and all the knowledge and expertise that traveled alongside it, the Spanish nuclear program was the product of a complex combination of geopolitical, scientific, technological, economic, and financial factors, as well as a stimulus for international relations, particularly with North American companies. The various agents involved in the construction of the Zorita nuclear power plant—experts, scientists, military officers, promoters, engineers, consultants, as well as energy consumers, both Spanish and American—show that this plant was a collective project, public and private, and impossible to understand without the international context. Thus, research on the history of radiation in Spain requires constant dialogue between the history of science and technology and international political, economic, and industrial histories of the second half of the twentieth century.

The acquisition of the reactor for Zorita was far more than a scientific and technical solution to meet national energy needs. Its installation created new disciplines, introduced changes in the landscape, created new industrial and administrative cartographies and, above all, influenced the collective imagination by showing that another model for the country was possible: a modern nation, internationally connected and with the technical capacity and political and business leadership capable of incorporating the latest atomic technologies. Purchase of the reactor and construction of the Zorita nuclear power plant participated in the construction of a new national and international narrative of the Francoist State that began in the 1960s.

The reactor not only brought atomic practices, methodologies, regulations and models to Spain. It also generated new political and business alliances that put technology at the center of Spanish political, industrial, and economic agendas. Zorita was more than an energy production center, it was also a production center for hybrid knowledge: physical, technical, political, and industrial. It was a facility in which to study the different scientific controversies that atomic practices created and the powerful role that scientific institutions responsible for safety standards acquired. Additionally, it served to analyze complex multinational power relationships, which were a product of nuclear diplomacy and its influence on the reconfiguration of industrial policies. In spite of the fact that in the 1960s reactor technology was still undergoing a maturation process, in the end Zorita served to reinforce the North American industry in Europe, which enabled the Americans to control the Western market for nuclear reactors.

Zorita incorporated a series of cutting-edge practices—not only in radiation safety—into Spanish science and technology. It also brought together power of the Franco dictatorship, the electrical companies, and the engineers in an alliance that announced the rise of technocratic Spain in the 1960s and early 1970s and made the site an important agent in the construction of the history of radiation in Spain.

## Acknowledgements

I would like to thank Juan Bros for granting me access to the Tecnatom Historical Archive. Being able to consult the material held by this private company was invaluable and I am extremely thankful. I am also grateful to Agustín Alonso for the documentation he provided and the conversations we had. This research has been carried out within the framework of the PID2019–106971 GB-I00 project funded by the Ministry of Science and Innovation of Spain.

## Footnotes

1 Three conferences were held to agree on criteria and radiological protection practices that were compatible in the different countries. The first was held in 1949 in Chalk River (Ontario). The second was held one year later in Harwell (England). The third and last Tripartite Conference was held in Harriman (New York) in 1953, where they drafted the final recommendations for new protection standards (Taylor 1984).

2 Cooperation Agreement between the Government of Spain and the Government of the United States of America on civilian uses of atomic energy. Washington, July 19, 1955. Archive of the Ministry of Foreign Affairs.

3 Between 1952 and 1982, 38 nuclear plants were planned in Spain but only ten—Zorita, Garoña, Vandellòs I and II, Almaráz I and II, Ascó I and II, Cofrentes, and Trillo II—were ultimately connected to the electricity grid (Rubio Varas and de la Torre 2017: 250–254).

4 It is beyond the scope of this article to delve deeper into what these “families” or groups, never parties, meant for the Franco regime. But it is indeed of interest to note that the different political sensitivities of each of them were used by the dictator to ensure he himself had an indisputable presence within the power structure.

5 There are records in the archives of the Nuclear Safety Council (CSN) of stays by Spanish researchers in French centers. One example is the visit by Manuel Perelló Palop, who worked with the reactor safety group (Groupe de Travails de Sécurité de Piles, GTPS) in the Department of Reactor Studies (Départament des Études de Piles, DEP) of the French Commissariat à l’Énergie Atomique (CEA). Upon his return to Spain, he was put in charge of planning and follow-up, with both the French technicians from EDF and the safety group from the JEN, for the different safety inspections needed by the UNGG technology installed at Vandellòs.

6 Law 25/1964 on Nuclear Energy, *Boletín Oficial del Estado (BOE*, Official State Bulletin), May 4, 1964, 107: 7544.

7 Order of 22 December 1959. *BOE*, 28-December-1959, 310: 16467–16468, whereby the Government Presidency, following recommendations made by the OEEC to all Member States—Spain became part of this organization in January 1958—issued regulations for protection from ionizing radiation.

8 Complete recommendations made by the OEEC in Acts of Organisation-OECD Legal Instruments. https://legalinstruments.oecd.org/public/doc/384/3b04967e-3485-4975-b729-3d531a92e738.pdf

9 The first prototypes of the TALGO train, designed by the Spanish engineer Alejandro Goicoechea, were built in the United States with financing from Banco Urquijo, majority owners of UEM, as has already been mentioned.

10 Economic sense of the Nuclear Power Plant requested by UEM. April 1962. Tecnatom Historical Archive.

11 In the United States, the reactors at Shippingport (1957), Dresden‑1 (1959), Yankee Rowe (1960), Saxton (1962), Indian Point (1962), Big Rock Point (1962) and ERR (1962) were under way. In Canada, NPD (1962). In France, G2 and G3 (1958–1959) and Chinon 1 (1962). In Italy, Latina was in operation (1962). The United Kingdom had Calder Hall (1956–1959), Chapelcross (1958–1959), DFR (1959), Berkeley (1961–1962), Bradwell (1961–1962) and WAGR (1962) operating. In Germany, Kahl was operating (1960). In Belgium, BR‑S (1962). And in the Soviet Union, APS (1954), Atoms on Wheels (1961) and Siberian (1958–1962).

12 A political fight broke out within the Franco regime between the more conservative and autarkic sectors and those that were committed to liberalizing the economy. The latter sectors, known as the technocrats, were led by people associated with Opus Dei and, as of 1957, held important posts in the government. But this internal fight in no way put into question the political foundations of the Franco regime.

13 However, the Ministry of Industry was not satisfied with the 40% local participation and increased the percentage in subsequent projects: in 1969 the Spanish National Electricity Plan revised local participation in nuclear projects until it reached 50% in 1972, 60% in 1975, and 75% in 1978 (Rubio-Varas & De la Torre 2017: xvi).

14 Reports show that participation of Spanish companies in the construction of Zorita reached 48% of the total. José Cabrera Nuclear Power Plant, 1970. Tecnatom Historical Archive.

15 Hydro-geological study for the future nuclear power plant in Zorita. Geological and Mining Institute of Spain. March 1963. Tecnatom Historical Archive.

16 Meteorological report on diffusion in Zorita. EPTISA (Industrial Technical Studies and Projects S.A.). November 1963. Tecnatom Historical Archive.

17 Related to population density in surrounding areas, the 1959 US AEC criteria suggested that all reactors be “several miles distant from the nearest town or city and for large reactors a distance of 10 to 20 miles from large cities” (Mazuzan & Walker 1985: 244). In this sense, the siting of Zorita totally meets the criteria established by the North American regulators.

20 To learn about the studies done on possible radioactivity accidents in more detail see, Unión Eléctrica Madrileña 1967: 5–56.

21 For studies done on mechanical accidents, see Unión Eléctrica Madrileña 1967: 76–80.

22 Unión Eléctrica Madrileña. Zorita Nuclear Power Plant. Program for zero power tests at the Zorita Nuclear Power Plant. Tecnatom Historical Archive.

23 Nuclear Test Reports. Nuclear Test Report no.1. Initial core loading. August 1968, pp. 2 and 3. Tecnatom Historical Archive.

26 No-Do, 1375B, shown December 23, 1968, http://www.rtve.es/filmoteca/no-do/not-1355/1486662/

27 The Tarapur power plant in India, which had also been acquired as a “turnkey,” did not become operational until 1969.
